# Assessing quality improvement capacity in primary care practices

**DOI:** 10.1186/s12875-019-1000-1

**Published:** 2019-07-25

**Authors:** Michael L. Parchman, Melissa L. Anderson, Katie Coleman, Le Ann Michaels, Linnaea Schuttner, Cullen Conway, Clarissa Hsu, Lyle J. Fagnan

**Affiliations:** 1 Kaiser Permanente Washington Health Research Institute, 1730 Minor Ave Ste 1600, Seattle, WA 98101 USA; 2 Oregon Rural Practice Research Network, Oregon Health Sciences University, Portland, OR USA; 3 VA Puget Sound Healthcare System, Seattle, WA USA

**Keywords:** Quality improvement, Primary health care, Patient care team, Process assessment

## Abstract

**Background:**

Healthy Hearts Northwest (H2N) is a study of external support strategies to build quality improvement (QI) capacity in primary care with a focus on cardiovascular risk factors: appropriate aspirin use, blood pressure control, and tobacco screening/cessation.

**Methods:**

To guide practice facilitator support, experts in practice transformation identified seven domains of QI capacity and mapped items from a previously validated medical home assessment tool to them. A practice facilitator (PF) met with clinicians and staff in each practice to discuss each item on the Quality Improvement Capacity Assessment (QICA) resulting in a practice-level response to each item. We examined the association between the QICA total and sub-scale scores, practice characteristics, a measure of prior experience with managing practice change, and performance on clinical quality measures (CQMs) for the three cardiovascular risk factors.

**Results:**

The QICA score was associated with prior experience managing change and moderately associated with two of the three CQMs: aspirin use (*r* = 0.16, *p* = 0.049) and blood pressure control (*r* = 0.18, *p* = 0.013). Rural practices and those with 2–5 clinicians had lower QICA scores..

**Conclusions:**

The QICA is useful for assessing QI capacity within a practice and may serve as a guide for both facilitators and primary care practices in efforts to build this capacity and improve measures of clinical quality.

**Trial registration:**

This trial is registered with www.clinicaltrials.gov Identifier# NCT02839382, retrospectively registered on July 21, 2016.

**Electronic supplementary material:**

The online version of this article (10.1186/s12875-019-1000-1) contains supplementary material, which is available to authorized users.

## Background

Although primary care practices are under increased pressure to improve care quality [1], little attention has been devoted to assessing and building the inherent ability of primary care practices to effectively conduct quality improvement activities [1–3]. This “QI capacity” has been defined as “…the ability of a primary care practice to engage in QI activities in a continuous and effective way.” [4] QI capacity requires that teams of individuals who work in primary care settings possess an in-depth knowledge of approaches to QI, have the requisite skills and commitment to apply that knowledge to clinical care improvement, and are supported by clinical information systems in that effort [5, 6]. A focus on building QI capacity/capability is one of the key characteristics of high performing health care organizations [7–9].

Smaller primary care practices comprise over half of all primary care practices in the U.S. [10] These smaller practices often struggle with improving their capacity to improve care quality [11]. Health care professionals in these settings are often not prepared to promote QI efforts or lead change activities [12, 13]. They also often lack the health IT infrastructure, training, resources and time required to build their capacity to do QI well [11, 14–17]. Given the shortage of primary care providers, clinicians in these practices often have large patient panels requiring a focus on day-to-day patient care activities rather than continually improving the way care is delivered. These factors have led to calls for external support infrastructure to build QI capacity in primary care practice settings [18–20].

In 2015, the Agency for Healthcare Research & Quality (AHRQ) launched the EvidenceNOW initiative across seven regional cooperatives in the United States [21–23] to expand our knowledge about how to effectively assist smaller primary care practices in building their QI capacity with a focus on improving cardiovascular disease (CVD) risk factors [23]. For each of these cooperatives AHRQ required the use of practice facilitation as a unifying strategy [18, 24] to provide the necessary external support. Healthy Hearts Northwest is the EvidenceNOW cooperative across Washington, Oregon and Idaho [25, 26]. To guide practices and PFs in this study, we needed to define the specific elements and behaviors that reflect the presence of QI capacity specific to improving clinical care quality in a practice. Such a self-assessment tool should provide important insights to PFs about the current state of QI capacity in the practice and inform both the practice and the PF about opportunities to build QI capacity. Without such a ‘roadmap’ to serve as a guide, practice PFs have noted challenges when supporting improvement in primary care practices [27].

Is such a self-assessment tool needed? A recent systematic review of approaches to evaluate QI capacity found substantial variation in approaches to measure or observe QI capacity and no organizational-level QI capacity building evaluations were found [6]. Specific to primary care, some tools and instruments exist that assess organizational characteristics more broadly associated with quality improvement [28–32] such as overall organizational maturity or development [33], organizational culture [34–36], or readiness for change [37, 38]. However, the concept of QI capacity is distinct from organizational culture or readiness [39]. QI capacity is reflected in the presence of capabilities and competencies possessed by a primary care team that directly impinge on the delivery of clinical care to a patient. It is an emergent property of the underlying knowledge, skills, culture and experience of those who work in a practice [40]. For example, the Change Process Capacity Questionnaire (CPCQ) was developed to measure organizational capability to manage change within a primary care practice setting [41]. It identifies a set of processes used to manage change (e.g. using opinion leaders) that might be useful in quality improvement initiatives. It was not explicitly developed to assess the presence of specific structural capabilities required to improve clinical performance on clinical quality measures, and a recent publication found no association between the CPCQ score and practice performance on clinical quality measures as would be expected from an assessment of QI capacity [42].

In response to this need, we identify and define a set of domains that would inform the selection of key activities practices could undertake to improve their QI capacity [43]. We map those domains to survey items and introduce the twenty-item Quality Improvement Capacity Assessment (QICA) tool used to guide PFs and practices in assessing and building their QI capacity.

## Methods

### Study setting and subjects

Healthy Hearts Northwest (H2N) took place within 209 smaller (10 or fewer clinicians) primary care practices across Washington State, Oregon and Idaho and the results of this randomized trial have been previously reported [26]. All practices received support from a practice facilitator (PF) for 15 months. In addition to PF, practices were randomized to receive educational outreach visits (based on principle of academic detailing), shared learning opportunities or both. The published trial results adhered to CONSORT guidelines for reporting clinical trials.

### Development of a QI capacity assessment instrument

To identify and define core concepts or “domains” that contribute to QI capacity two members of the study team (KC, LM) along with two outside experts with considerable prior experience in patient centered medical home transformation efforts was convened. They first reviewed key articles in the literature related to the definitions and characteristics of QI capacity [5, 44], principles of transformation to a patient-centered medical home [45–49], and elements of the chronic care model [50, 51] [52]. .After reviewing the literature and discussing their prior medical home transformation experience, the experts used a nominal group process to nominate and build consensus about the domains [53]. A key consideration in selecting a domain was their assessment that a practice could engage in activities with support from a practice facilitator that would improve that domain within the planned 15-months of practice support. In addition, because the study was focused in improving CVD risk factors with support from a PF, there was a focus on domains that were felt to directly impact QI capacity in a manner that would improve performance on clinical quality measures of these risk factors [43]. These domains were:

1. Embed clinical evidence into daily work to guide how care is delivered to patients
2. Utilize data to understand and improve clinical performance measures
3. Establish a regular QI process involving cross-functional teams
4. Identify at-risk patients through pro-active population management for outreach
5. Define roles and responsibilities across the team to improve care
6. Deepen patient self-management support to improve clinical outcomes
7. Link patients to resources outside of the clinic to support patients

To assist both practices and their PF in assessing their current state of QI capacity and guiding efforts to improve, a self-assessment survey was developed by identifying items relevant to each of the seven change concepts. The Safety Net Medical Home Initiative had previously validated the 36-item Patient-Centered Medical Home Assessment (PCMH-A) tool for medical home transformation [54]. Since this instrument was intended to measure areas where practice change would result in improving care delivery, and because it was used by practice facilitators to support practice change, it was identified as a promising source of items. Study team members (MLP, KC) reviewed the 36 items in the PCMH-A independently, and agreed that 19 aligned well with the seven change concepts described above. A twentieth item was created by study team members (MLP, KC) to assess the availability of a standard method or tool to stratify patients by level of clinical risk. This new item was included with three other items to help assess the fourth domain listed above, identification of at-risk patients. Consistent with the PCMH-A instrument [54], and with the more widely used and validated Assessment of Chronic Illness Care (ACIC) instrument [27, 55], each item on the QICA is rated on a 1 to 12 scale with higher scores indicating higher QI capacity (see Additional file 1). The 20 items were grouped into seven subscales corresponding to the change concepts identified above.

### Measures and data collection

During a face-to-face visit by the PF at the start of the study intervention, the QICA survey was completed individually by clinicians and staff in each H2N practice. With the PF present, team members discussed their response to each item and came to a consensus on a practice-level response. These meetings took place between December 2015 and July of 2016. Prior to this meeting, practice managers completed a survey describing practice characteristics, which included the Change Process Capacity Questionnaire (CPCQ) [41]. In addition, each practice was asked to submit clinical quality measures (CQMs) data derived from the electronic health record on three CVD risk factors in the year prior to completing the QICA: appropriate aspirin use, blood pressure control, and tobacco screening/cessation [56, 57]. After the in-person visit to facilitate completion of the QICA, PFs kept detailed field notes describing the encounter.

### Analysis

We first described practice characteristics, and summarized baseline QICA scores and clinic performance on each CQM. We then used Kruskal-Wallis tests to assess the association between total QICA score and practice characteristics including practice size, ownership and location (rural v. urban). To examine the reliability of the QICA, we estimated Cronbach’s alpha for the total scale and each subscale. We assessed scale validity by reporting the Spearman correlation between the total score and subscale scores, with the CPCQ total score, and practice performance on each of the three CQMs in the year prior to administering the QICA survey.

## Results

The QICA was completed by 202 of the 209 enrolled practices. Of these, 178 (88.1%) provided data on the CPCQ and 192 (95.0%) reported on one or more of the CVD risk factor CQMs. Practice characteristics, QICA scores and CQMs are shown in Table 1. Of the seven change domains, practices scored the lowest on utilizing data, having a regular QI process, and identifying at-risk patients. Practices in rural locations, and those with 2–5 clinicians compared to solo or larger group practices had lower total QICA scores (Table 2).

**Table 1:** Practice Characteristics and QICA Scores(*n* = 202)

Location, n (%)	
Rural	90 (44.5)
Urban	112 (55.5)
Number of clinicians, n (%)	
One (solo)	37 (18.3)
2 to 4	107 (53.0)
5 or more	58 (28.7)
Organizational type, n(%)	
FQHC	22 (10.9)
Health/Hospital system	79 (39.1)
IHS/Tribal	10 (5.0)
Independent	91 (45.1)
Insurance type, mean percent (SD)*	
Medicare	24.5 (16.8)
Medicaid	24.4 (20.1)
Dual (Medicare and Medicaid)	3.6 (6.8)
Commercial	36.2 (22.6)
Uninsured	7.5 (12.9)
Other	3.8 (9.0)
Blood Pressure Clinical Quality Measure (CQM)	
Valid CQM data submitted, n(%)	189 (93.6)
CQM, mean (SD)	62.0 (12.7)
CQM, median (IQR)	61.6 (16.1)
CQM ≥70%, n(%)	44 (23.2)
Aspirin CQM	
Valid CQM data submitted, n(%)	155 (76.7)
CQM, mean (SD)	67.4 (16.4)
CQM, median (IQR)	69.4 (21.9)
CQM ≥70%, n(%)	72 (46.5)
Smoking CQM	
Valid CQM data submitted, n(%)	162 (80.2)
CQM, mean (SD)	73.1 (24.0)
CQM, median (IQR)	80.7 (21.4)
CQM ≥70%, n(%)	115 (71.0)
Quality Improvement Capacity Assessment (QICA)	Mean (SD)
Total Score, 20-items	6.55 (1.49)
HLC1: Embed clinical evidence	7.00 (2.37)
HLC2: Utilize data	5.15 (2.56)
HLC3: QI process	5.20 (2.23)
HLC4: Identify at-risk patients	5.64 (2.01)
HLC5: Team roles and responsibility	7.00 (1.90)
HLC6: Self-management support	7.45 (1.83)
HLC7: Linkage to resources	8.40 (1.74)

*Missing Insurance type, *n* 42 practices, *CQM* Clinical quality measure, *FQHC* Federally qualified health center, *IHS* Indian health service, *IQR* Interquartile range, *SD* Standard deviation

**Table 2:** Association of QICA with Practice Characteristics

	QICA Score	
	Mean (sd)	*P*-value*
Location		0.009
Rural	6.20 (1.41)	
Urban	6.83 (1.51)	
Number of clinicians		0.007
One (solo)	6.92 (1.35)	
2 to 5	6.24 (1.47)	
6 or more	6.89 (1.52)	
Organizational type		0.97
FQHC	6.62 (1.38)	
Health/Hospital system	6.53 (1.43)	
IHS/Tribal	6.29 (0.71)	
Independent	6.58 (1.65)	

*p-value from Kruskal-Wallis test

The Cronbach’s alpha was 0.90 for the total score and ranged from 0.62 to 0.87 for the domain subscales. (Table 3) All QICA sub-scores, as well as the total QICA score were significantly related to the practice’s CPCQ score. The QICA total score was associated with two of the three clinical quality measures: the proportion with adequate blood pressure control (*r* = 0.18, *p* = 0.013) and the proportion with appropriate aspirin use (*r* = 0.16, *p* = 0.049). Five of the seven QICA domain sub-scales were associated with at least one of the three CQMs.

**Table 3:** Correlation of the QICA with CPCQ and CQMs

QICA Item, total, or Domain sub-scale score	Reliability	Validity (Spearman correlation)			
	Cronbach’s alpha	CPCQ Score	CQM: Blood pressure	CQM: Aspirin	CQM: Smoking
Total Score, 20-item QICA	0.90	**0.351**	**0.180**	**0.158**	0.107
Domain sub-scales					
Embed clinical evidence	–	**0.280**	0.113	0.080	0.151
Utilize data	0.81	**0.301**	0.064	0.134	**0.186**
QI process	0.87	**0.278**	**0.148**	0.101	0.062
Identify at-risk patients	0.77	**0.351**	0.058	**0.170**	0.103
Team roles and responsibility	0.65	**0.172**	0.124	0.108	0.047
Self-management support	0.77	**0.194**	**0.191**	0.111	0.058
Linkage to resources	0.62	**0.322**	**0.190**	0.000	0.070

(Bold font indicates *p* < 0.05)

## Discussion

These results suggest that the QICA is associated both with a practices’ prior experience with specific change strategies (as measured by the CPCQ) and their current level of clinical performance as assessed by two of the three CVD CQMs collected for this study. These findings provide partial support for the “criterion validity” of the QICA, the degree to which a measure is associated with an outcome. Overall, practices scored near the middle of the range of possible scores on the total QICA score, 6.55 (S.D. 1.49), out of a possible range of 1 to 12, with no evidence of either floor or ceiling effects. Scores on utilizing data to improve, establishing a regular QI process, and identifying at-risk patients for outreach were lower than other QI capacity domains. These three domains represent what some consider to be core QI competencies [43], reflect significant opportunities for development of their QI capacity, and support prior observations that smaller primary care practices struggle with building their QI capacity [58]..

The total QICA score was moderately associated with two of the three CQMs. In addition to QI capacity, it is possible that other inner setting characteristics of the clinic, its patient population, and other external/contextual factors play an important role in predicting performance on CQMs [59]. For example, some practices may have had external incentives to improve performance, financial or otherwise, that interacted with their QI capacity to predict their performance. QI capacity is also dependent on the interrelationships between QI training; individual capability and capacity; opportunities to apply QI skills; exposure to further coaching/support in the workplace; a supportive organizational culture and leadership; and access to resources [6]. Cohen and colleagues highlighted the role of a complex interaction of internal and external factors on implementing improvement strategies in primary care [60]. Those factors include motivations of practice members to improve, the resources available, external influences on improvement options, and the perspective of practice members about where opportunities for improvement exist. Ultimately, although the domains represented in the QICA reflect the internal practice setting and may be necessary for improvement to occur, they may not be sufficient to fully explain current performance on CQMs.

In addition to these limitations it is worth noting that some practices may have scored themselves higher on some items on the QICA survey because they did not fully understand what the highest state or level would look like in their setting, or their practice’s true ability in relation to this target highest level. This overestimation may have been more common in practices with less QI experience and less appreciation for the doing the work of QI. The investigators are cognizant of the possibility that practices with higher QI capacity had employed that capacity to improve other measures of care quality but had not focused improvement efforts on their performance on the cardiovascular risk factors reported in this study. For example, some practices may have done prior work on improving performance on measures of cancer screening or immunizations in response to external influences, work that improved their QI capacity, but had never engaged in work to improve the cardiovascular CQMs measured here.

## Conclusion

The QICA is useful for assessing QI capacity within a practice and may serve as a guide for both facilitators and primary care practices in efforts to improve this capacity and improve performance on clinical quality measures.

## Additional file

Additional file 1: Quality Improvement Change Assessment. (PDF 343 kb)

## Data Availability

The datasets used and/or analyzed during the current study are available from the corresponding author on reasonable request.

## Abbreviations

AHRQ: Agency for Healthcare Research & Quality
CQM: Clinical Quality Measure
CVD: cardiovascular disease
H2N: Healthy Hearts Northwest study
PF: Practice Facilitator
QI: Quality Improvement
QICA: Quality Improvement Capacity Assessment
