# Supplementary material for: Assessing quality improvement capacity in primary care practices
Source: BMC Fam Pract. 2019 Jul 25;20:103. doi: 10.1186/s12875-019-1000-1 (PMC6657073; doi:10.1186/s12875-019-1000-1)
Supplement: Supplementary file 1 — Quality Improvement Change Assessment. (PDF 343 kb) [file 12875_2019_1000_MOESM1_ESM.pdf]

HEALTHY HEARTS NORTHWEST
An EvidenceNOW Project

Quality Improvement Change Assessment

HLC 1: Embed clinical evidence on ABCS into daily work to guide care for patients

Items	Level D	Level C	Level B	Level A
1. Comprehensive, guideline-based information on prevention or chronic illness treatment	…is not readily available in practice.	…is available but does not influence care.	…is available to the team and is integrated into care protocols and/or reminders.	…guides the creation of tailored, individual-level data that is available at the time of the visit.
	1 2 3	4 5 6	7 8 9	10 11 12

HLC 2: Utilize reliable, robust data to understand and improve ABCS measures

Items	Level D	Level C	Level B	Level A
2. Performance measures	…are not available for the clinical site.	…are available for the clinical site, but are limited in scope.	…are comprehensive—including clinical, operational, and patient experience measures—and available for the practice, but not for individual providers.	…are comprehensive—including clinical, operational, and patient experience measures—and fed back to individual providers.
	1 2 3	4 5 6	7 8 9	10 11 12
3. Reports on care processes or outcomes of care	…are not routinely available to practice teams.	…are routinely provided as feedback to practice teams but not reported externally.	…are routinely provided as feedback to practice teams, and reported externally (e.g., to patients, other teams or external agencies) but with team identities masked.	…are routinely provided as feedback to practice teams, and transparently reported externally to patients, other teams and external agencies.
	1 2 3	4 5 6	7 8 9	10 11 12

This project is supported by grant number R18HS023908 from the Agency for Healthcare Research and Quality
This is an abbreviated version of the Safety Net Medical Home Initiative, Patient-Centered Medical Home Assessment Version 4.0. Seattle, WA: The MacColl Center for Health Care Innovation at Group Health Research Institute and Qualis Health; September 2014 (http://www.safetynetmedicalhome.org/sites/default/files/PCMH-A.pdf)

Quality Improvement Change Assessment

HLC 3: Establish a regular QI Process Involving cross-functional teams

Items	Level D	Level C	Level B	Level A
4. The responsibility for conducting quality improvement activities	…is not assigned by leadership to any specific group.	…is assigned to a group without committed resources.	…is assigned to an organized quality improvement group who receive dedicated resources.	…is shared by all staff, from leadership to team members, and is made explicit through protected time to meet and specific resources to engage in QI.
	1 2 3	4 5 6	7 8 9	10 11 12
5. Quality improvement activities	…are not organized or supported consistently.	…are conducted on an ad hoc basis in reaction to specific problems.	…are based on a proven improvement strategy in reaction to specific problems.	…are based on a proven improvement strategy and used continuously in meeting organizational goals.
	1 2 3	4 5 6	7 8 9	10 11 12
6. Quality improvement activities are conducted by	…a centralized committee or department.	…topic specific QI committees.	…all practice teams supported by a QI infrastructure.	…practice teams supported by a QI infrastructure with meaningful involvement of patients and families.
	1 2 3	4 5 6	7 8 9	10 11 12

This project is supported by grant number R18HS023908 from the Agency for Healthcare Research and Quality
This is an abbreviated version of the Safety Net Medical Home Initiative, Patient-Centered Medical Home Assessment Version 4.0. Seattle, WA: The MacColl Center for Health Care Innovation at Group Health Research Institute and Qualis Health; September 2014 (http://www.safetynetmedicalhome.org/sites/default/files/PCMH-A.pdf)

Quality Improvement Change Assessment

HLC 4: Identify at-risk patients for prevention outreach

Items	Level D	Level C	Level B	Level A
7. Registry or panel-level data	…are not available to assess or manage care for practice populations.	…are available to assess and manage care for practice populations, but only on an ad hoc basis.	…are regularly available to assess and manage care for practice populations, but only for a limited number of diseases and risk states.	…are regularly available to assess and manage care for practice populations, across a comprehensive set of diseases and risk states.
	1 2 3	4 5 6	7 8 9	10 11 12
8. Registries on individual patients	…are not available to practice teams for pre-visit planning or patient outreach.	…are available to practice teams but are not routinely used for pre-visit planning or patient outreach.	…are available to practice teams and routinely used for pre-visit planning or patient outreach, but only for a limited number of diseases and risk states.	…are available to practice teams and routinely used for pre-visit planning and patient outreach, across a comprehensive set of diseases and risk states.
	1 2 3	4 5 6	7 8 9	10 11 12
9. A standard method or tool(s) to stratify patients by risk level	…is not available.	…is available but not consistently used to stratify all patients.	…is available and is consistently used to stratify all patients but is inconsistently integrated into all aspects of care delivery.	…is available, consistently used to stratify all patients, and is integrated into all aspects of care delivery.
	1 2 3	4 5 6	7 8 9	10 11 12

This project is supported by grant number R18HS023908 from the Agency for Healthcare Research and Quality
This is an abbreviated version of the Safety Net Medical Home Initiative, Patient-Centered Medical Home Assessment Version 4.0. Seattle, WA: The MacColl Center for Health Care Innovation at Group Health Research Institute and Qualis Health; September 2014 (http://www.safetynetmedicalhome.org/sites/default/files/PCMH-A.pdf)

Quality Improvement Change Assessment

Items	Level D	Level C	Level B	Level A
10. Visits	…largely focus on acute problems of patient.	…are organized around acute problems but with attention to ongoing illness and prevention needs if time permits.	…are organized around acute problems but with attention to ongoing illness and prevention needs if time permits. The practice also uses subpopulation reports to proactively call groups of patients in for planned care visits.	…are organized to address both acute and planned care needs. Tailored guideline-based information is used in team huddles to ensure all outstanding patient needs are met at each encounter.
	1 2 3	4 5 6	7 8 9	10 11 12

HLC 5: Define roles and responsibilities (tasks) across the care team to identify and manage ABCs

Items	Level D	Level C	Level B	Level A
11. Non-physician practice team members	…play a limited role in providing clinical care.	…are primarily tasked with managing patient flow and triage.	…provide some clinical services such as assessment or self-management support.	…perform key clinical service roles that match their abilities and credentials.
	1 2 3	4 5 6	7 8 9	10 11 12
12. The practice	…does not have an organized approach to identify or meet the training needs for providers and other staff.	…routinely assesses training needs and ensures that staff are appropriately trained for their roles and responsibilities.	…routinely assesses training needs, ensures that staff are appropriately trained for their roles and responsibilities, and provides some cross training to permit staffing flexibility.	…routinely assesses training needs, ensures that staff are appropriately trained for their roles and responsibilities, and provides cross training to ensure that patient needs are consistently met.
	1 2 3	4 5 6	7 8 9	10 11 12

This project is supported by grant number R18HS023908 from the Agency for Healthcare Research and Quality
This is an abbreviated version of the Safety Net Medical Home Initiative, Patient-Centered Medical Home Assessment Version 4.0. Seattle, WA: The MacColl Center for Health Care Innovation at Group Health Research Institute and Qualis Health; September 2014 (http://www.safetynetmedicalhome.org/sites/default/files/PCMH-A.pdf)

Quality Improvement Change Assessment

Items	Level D	Level C	Level B	Level A
13. Care Plans	…are not routinely developed or recorded.	…are developed and recorded but reflect providers' priorities only.	…are developed collaboratively with patients and families and include self-management and clinical goals, but they are not routinely recorded or used to guide subsequent care.	…are developed collaboratively, include self-management and clinical management goals, are routinely recorded, and guide care at every subsequent point of service.
	1 2 3	4 5 6	7 8 9	10 11 12
14. Clinical care management services for high-risk patients	…are not available.	…are provided by external care managers with limited connection to practice.	…are provided by external care managers who regularly communicate with the care team.	…are systematically provided by the care manager functioning as a member of the practice team, regardless of location.
	1 2 3	4 5 6	7 8 9	10 11 12

HLC 6: Deepen patient self-management support for action planning around ABCs

Items	Level D	Level C	Level B	Level A
15. Assessing patient and family values and preferences	…is not done.	…is done, but not used in planning and organizing care.	…is done and providers incorporate it in planning and organizing care on an ad hoc basis.	…is systematically done and incorporated in planning and organizing care.
	1 2 3	4 5 6	7 8 9	10 11 12

This project is supported by grant number R18HS023908 from the Agency for Healthcare Research and Quality
This is an abbreviated version of the Safety Net Medical Home Initiative, Patient-Centered Medical Home Assessment Version 4.0. Seattle, WA: The MacColl Center for Health Care Innovation at Group Health Research Institute and Qualis Health; September 2014 (http://www.safetynetmedicalhome.org/sites/default/files/PCMH-A.pdf)

Quality Improvement Change Assessment

Items	Level D	Level C	Level B	Level A
16. Involving patients in decision-making and care	…is not a priority.	…is accomplished by provision of patient education materials or referrals to classes.	…is supported and documented by practice teams.	…is systematically supported by practice teams trained in decision-making techniques.
	1 2 3	4 5 6	7 8 9	10 11 12
17. Self-management support	…is limited to the distribution of information (pamphlets, booklets).	…is accomplished by referral to self-management classes or educators.	…is provided by goal setting and action planning with members of the practice team.	…is provided by members of the practice team trained in patient empowerment and problem-solving methodologies.
	1 2 3	4 5 6	7 8 9	10 11 12

HLC 7: Develop robust linkages to smoking cessation, CDSMP and other evidence-based community resources

Items	Level D	Level C	Level B	Level A
18. Test results and care plans	…are not communicated to patients.	…are communicated to patients based on an ad hoc approach.	… are systematically communicated to patients in a way that is convenient to the practice.	…are systematically communicated to the patients in a variety of ways that are convenient to patients.
	1 2 3	4 5 6	7 8 9	10 11 12

This project is supported by grant number R18HS023908 from the Agency for Healthcare Research and Quality
This is an abbreviated version of the Safety Net Medical Home Initiative, Patient-Centered Medical Home Assessment Version 4.0. Seattle, WA: The MacColl Center for Health Care Innovation at Group Health Research Institute and Qualis Health; September 2014 (http://www.safetynetmedicalhome.org/sites/default/files/PCMH-A.pdf)

Quality Improvement Change Assessment

Items	Level D	Level C	Level B	Level A
19. Patients in need of specialty care, hospital care, or supportive community- based resources	…cannot reliably obtain needed referrals to partners with whom the practice has a relationship.	…obtain needed referrals to partners with whom the practice has a relationship.	…obtain needed referrals to partners with whom the practice has a relationship and relevant information is communicated in advance.	…obtain needed referrals to partners with whom the practice has a relationship, relevant information is communicated in advance, and timely follow-up after the visit occurs.
	1 2 3	4 5 6	7 8 9	10 11 12
20. Linking patients to supportive community- based resources	…is not done systematically.	…is limited to providing patients a list of identified community resources in an accessible format.	…is accomplished through a designated staff person or resource responsible for connecting patients with community resources.	…is accomplished through active coordination between the health system, community service agencies and patients and accomplished by a designated staff person.
	1 2 3	4 5 6	7 8 9	10 11 12

This project is supported by grant number R18HS023908 from the Agency for Healthcare Research and Quality
This is an abbreviated version of the Safety Net Medical Home Initiative, Patient-Centered Medical Home Assessment Version 4.0. Seattle, WA: The MacColl Center for Health Care Innovation at Group Health Research Institute and Qualis Health; September 2014 (http://www.safetynetmedicalhome.org/sites/default/files/PCMH-A.pdf)
